# Editorial: Dysbiosis and microbiome modulation in cancer prevention and therapy

**DOI:** 10.3389/fonc.2026.2006794

**Published:** 2026-10-19

**Authors:** Rohini Ravindran Nair, Ehud Wainstein, Suresh Singh Yadav

**Affiliations:** 1 Department of Medical Biotechnology, Gujarat Biotechnology University (GBU), Gandhinagar, Gujarat, India; 2 Yitzhak Shamir Medical Center (formerly Assaf Harofeh Medical Center), Be'er Ya'akov, Israel; 3 Department of Molecular Biology & Biochemistry, Guru Nanak Dev University (GNDU), Amritsar, Punjab, India

**Keywords:** cancer, cancer therapy, dysbiosis, gut microbiome, microbiome modulation

The human microbiome, which consists of billions of microorganisms living mostly in the gastrointestinal tract as well as the mouth, skin, and genitourinary tract, has become a key element of cancer biology. Physiologically, the microorganisms exist in mutual equilibrium with their host, taking part in nutrient metabolism, educating the immune system, and protecting from pathogens. The microbial balance may be broken because of diet, antibiotics, infections, chronic inflammation, and lifestyle factors such as alcohol or smoking, thus causing dysbiosis. In reality, dysbiosis is not a state of microorganisms, but is rather defined by loss of symbiotic microflora, proliferation of pathobionts, and decreased microbial diversity and redundancy. Those changes lead to the development of pathological conditions in various tissues and have been linked to adverse processes such as cancer progression and resistance to therapy.

Dysbiosis can promote carcinogenesis through several mechanisms. Disruption of the mucus layer, epithelial tight junctions, and antimicrobial defences increases microbial penetration into the epithelium, activating Toll-like and NOD-like receptors and downstream inflammatory pathways, including NF-κB and cGAS-STING. This promotes chronic inflammation characterised by increased TNF-α, IL-6, and IL-17, as well as the recruitment of myeloid-derived suppressor cells and regulatory T cells. In addition, some bacteria can directly cause DNA damage and activate oncoproteins. - Indeed, pks+ *Escherichia coli* producing colibactin causes DNA cross-links and specific mutations that are observed in colorectal cancer patients, while enterotoxigenic *Bacteroides fragilis* cleaves E-cadherin. Finally, dysbiosis also affects host metabolism, causing the reduction of protective short-chain fatty acids like butyrate and accumulation of secondary bile acids, lactate, and polyamines that support tumorigenesis.

These mechanisms are different for each kind of cancer. *Fusobacterium nucleatum* has been associated with promoting colorectal cancer development through FadA-mediated E-cadherin/β-catenin signaling and Fap2-mediated activation of TIGIT receptor expressed on immune cells, helping the cancer to evade the immune system. Similar associations can be found in *Fusobacterium nucleatum* and *Porphyromonas gingivalis*, which can contribute to head and neck carcinogenesis by means of epithelial disruption, inflammation, and immune manipulation. For instance, the latter has been shown to induce overexpression of immune checkpoints like PD-L1 in the oral epithelium and cancer cells, possibly helping with immune escape. The microbiome also plays a role in the efficiency, toxicity and resistance to cancer treatment. Commensals producing short-chain fatty acids (SCFAs) and some metabolites like inosine or indole-3-aldehyde contribute to immune checkpoint inhibitors’ efficiency, whereas dysbiosis caused by antibiotics correlates with worse treatment outcomes. β-glucuronidase enzymes of gut microbes convert irinotecan to its toxic metabolite, causing diarrhoea, and γ-proteobacteria destroy gemcitabine, making chemotherapy less effective. The β-glucuronidase can deconjugate the estrogen glucuronide metabolites from the liver and produce active estrogens, which are recycled via enterohepatic recirculation. Thus, elevated β-glucuronidase enzyme activity of gut bacteria could lead to increased exposure of breast cells to active estrogens and promote estrogen receptor-positive (ER+) breast cancer. Moreover, microbial composition correlates with radiotherapy-induced toxicity and systemic antitumor activity.

The facts mentioned above have led to attempts to alter the microbiome using dietary interventions, prebiotics, probiotics, live biotherapeutics, and faecal transplants in order to improve therapeutic efficacy and reduce potential toxicities of treatment. However, the clinical translation is complicated by high individual variability among patients, differences in methodology of microbiome studies, and a lack of understanding of microorganisms’ function. The perception of dysbiosis as a set of alterations at the level of organisms and pathways is necessary for developing targeted microbiome-based methods for cancer prevention and therapy.

All of the papers published on the topic, taken together, support one common idea: the microbiome does not act as a mere bystander or a simple driver of cancer development but as a complex distributed network. When this network is disrupted, a condition known as dysbiosis, it can influence inflammation, genotoxicity, immune evasion, and metabolism throughout cancer initiation and progression.

Lin et al. discuss that the focus is no longer on the organisms, but rather on the communication between the oral-gut axis microorganisms. Head and neck cancer (HNC) is increasingly linked to oral and gut microbiota dysbiosis, in addition to established risk factors such as tobacco and viral infections. The oral-gut axis may promote HNC through microbial translocation, altered metabolites, chronic inflammation, immune suppression, and activation of oncogenic pathways. Targeting the oral-gut axis with probiotics, antimicrobial peptides, and microbiome-based interventions could potentially reduce treatment-related mucosal injury and improve therapeutic responses. Further, Li et al. propose a novel gut-thyroid axis, in which *Terrisporobacter* may promote papillary thyroid carcinoma (PTC) progression through NTRK1/TRK signaling and immune suppression, highlighting the gut microbiome as a potential regulator of papillary thyroid cancer progression. Al Shareef et al. investigated the prostate cancer tissue microbiota and showed that microbial diversity was significantly higher in prostate cancer samples compared to benign prostatic hyperplasia tissues, and had more variation in their microbial composition. Among the identified bacterial genera, *Methylobacterium* was more abundant in prostate cancer, and this association remained consistent even after adjusting for other clinical variables. Dixit et al.’s comprehensive review provides a good “two-hit” approach: the first dysbiosis hit creates a permissive and barrier-deficient microenvironment, while the second hit - genotoxicity, persistent inflammation, immune escape, etc. drives malignant transformation. Author suggested that, however, clinical translation remains challenging because of individual variability, mechanistic complexity, and lack of standardisation. These challenges underscore the need for personalised, microbiome-informed approaches to cancer prevention and therapy.

Three common points appear: methodological caution, increased mechanistic specificity and standardisation for clinical applications. The field is moving away from general “good vs bad” notions of the microbiome to specific pathways like FadA/E-cadherin, Fap2/TIGIT, colibactin/DNA damage and SCFA signalling. The challenge is now harmonisation of sampling, sequencing, functional validation, donor selection and regulatory aspects. Future progress in this field will probably use integration of multi-omic approaches and mechanisms rather than the discovery of new organisms.

